# Rigid Basepair Monte Carlo Simulations of One-Start and Two-Start Chromatin Fiber Unfolding by Force

**DOI:** 10.1016/j.bpj.2018.10.007

**Published:** 2018-10-11

**Authors:** Babette E. de Jong, Thomas B. Brouwer, Artur Kaczmarczyk, Bert Visscher, John van Noort

**Affiliations:** 1 Huygens-Kamerlingh Onnes Laboratory, Leiden Institute of Physics, Leiden University, Leiden, The Netherlands

## Abstract

The organization of chromatin in 30 nm fibers remains a topic of debate. Here, we quantify the mechanical properties of the linker DNA and evaluate the impact of these properties on chromatin fiber folding. We extended a rigid basepair DNA model to include (un)wrapping of nucleosomal DNA and (un)stacking of nucleosomes in one-start and two-start chromatin fibers. Monte Carlo simulations that mimic single-molecule force spectroscopy experiments of folded nucleosomal arrays reveal different stages of unfolding as a function of force and are largely consistent with a two-start folding for 167 and one-start folding for 197 nucleosome repeat length fibers. The major insight is that nucleosome unstacking and subsequent unwrapping is not necessary to obtain quantitative agreement with experimental force extension curves up to the overstretching plateau of folded chromatin fibers at 3–5 pN. Nucleosome stacking appears better accommodated in one-start than in two-start conformations, and we suggest that this difference can compensate the increased energy for bending the linker DNA. Overall, these simulations capture the dynamic structure of chromatin fibers while maintaining realistic physical properties of the linker DNA.

## Introduction

The folding of chromatin fibers remains elusive despite more than three decades of research (1, 2, 3, 4, 5, 6, 7). It is an important topic in biology because transcription regulation, and perhaps all processes involving DNA, are regulated by chromatin (8). Post-translational modifications (PTMs) of both the histone tails and the globular parts of the histones have proven to be the hallmarks of activity on DNA that is wrapped around histone cores (9). Some of these PTMs can be linked to structural features of the chromatin. For example, the acetylation of H3K56, which is indicative of transcriptionally active chromatin, increases the unwrapping of nucleosomal DNA (10). Acetylation of H4K16, also prominent in transcriptionally active euchromatin, has been shown to affect fiber folding (11, 12, 13). Other modifications, like methylation of H3K9, induce the transition from euchromatin to more condensed heterochromatin, mediated by heterochromatin protein 1 (HP1) (14). Next to direct structural changes to the chromatin, PTMs regulate the binding of remodeling factors that add or remove other PTMs or reposition, assemble, and/or disassemble nucleosomes. Although significant progress has been made in the structural biology of single nucleosomes and their complexes with other factors (7), a consensus on the structure of higher-order folded chromatin fibers is dearly lacking.

High-resolution crystallography (15) and, more recently, single-particle electron microscopy (EM) (16) of reconstituted fibers clearly point to a two-start organization. Attractive interactions between odd and even nucleosomes in such fibers induce the formation of a double superhelix of stacked nucleosomes. Attempts to resolve the structure of native chromatin fibers by EM, super-resolution light microscopy, or diffraction techniques, however, generally yield less order (17, 18, 19), if any, suggesting the absence of higher-order structure in vivo. Because the positions of the nucleosomes on the DNA are far from regular in vivo, it is hard to avoid disorder induced by variations in the lengths of the linker DNA in natively assembled chromatin fibers. To avoid such positional disorder of nucleosomes in vitro, chromatin fibers are typically reconstituted on tandem repeats of the strong Widom 601 nucleosome positioning sequence, which allows for perfect control of the nucleosome repeat length (NRL) (20). Systematic variation of the linker length in steps of 10 basepairs (bp) and subsequent reconstitution, folding, and EM inspection of highly condensed fibers with linker histone H5 yielded a diameter of 33 nm that was independent of linker length (20). This observation is incompatible with a two-start helix and suggests a one-start structure for NRLs between 177 and 207 bp. Unfortunately, both the contrast and the relative disorder of the nucleosomes has prevented elucidation of the path of the DNA in most of the larger NRL fibers, leaving a robust structural interpretation in the open.

Single-molecule force spectroscopy provides a unique method to probe and manipulate the extension of folded chromatin fibers under physiological conditions and can therefore contribute to a better structural understanding of the 30 nm chromatin fiber. In previous studies, we have reported force-extension data of 167 and 197 NRL fibers (21, 22, 23), which feature distinctive low-force unfolding characteristics that we have interpreted as resulting from two-start and one-start folding, respectively. However, this interpretation was challenged, suggesting that the data could equally well be explained by the gradual unwrapping of nucleosomal fibers without stacking interactions (24). We observed that the force-extension curves with and without linker histones were indistinguishable up to the overstretching transition, which increased to forces above 5 pN when linker histones were present (22), raising the question of how condensed chromatin fibers are organized at forces below the overstretching transition. Here, we study the unfolding of three alternative structures: nonstacked, one-start-stacked, or two-start-stacked chromatin fibers. Therefore, a more quantitative, physics-driven structural model, rather than qualitative arguing. is required to interpret the force-extension data.

A large variety of early models for chromatin structure focused on experimentally observed geometric constraints to capture the high level of condensation achieved by chromatin (25, 26, 27, 28, 29, 30, 31). Many of these models, however, cannot easily be adapted to include forced unfolding of chromatin fibers, though topological restrictions might exclude some of them. Full-atom molecular dynamics, on the other hand, would give a detailed structural insight, but the timescale of the simulations differs by many orders of magnitude with the experimental results. Coarse-grained Monte Carlo (MC) models have successfully reproduced several features of the experimental force-extension data. Rippe et al. modeled chromatin fibers consisting of nucleosome and DNA beads in combination with a nucleosome-nucleosome interaction potential (32). The typical chromatin overstretching plateau at 3–5 pN was attributed to breaking nucleosome-nucleosome interactions. Collepardo-Guevara and Schlick presented a model containing more flexible linker DNA in combination with electrostatic interactions arising from charges that were distributed along the coarse-grained DNA and histone proteins (28). Their model could also reproduce the typical low-force overstretching plateau when nucleosome-nucleosome stacking interactions, typically in two-start fibers, were broken. Recently, Norouzi and Zhurkin (33) employed a rigid basepair model in combination with geometrical constraints to compute the DNA trajectory of the linker DNA in two-start fibers with high quantitative agreement. Again, the low-force overstretching plateau is reproduced but now in more detail. It is proposed that gradual unwrapping of nucleosomal DNA can account for the linear stretching region between 1 and 3 pN before the overstretching plateau sets in.

Although it may seem that current coarse-grained models suffice to explain experimental force-extension curves, there are several features in the collective data set that cannot be captured in the above-described two-start geometry and that are better explained in a one-start geometry (21, 23, 34). In this work, we first reiterate our experimental findings on pulling individual folded chromatin fibers and highlight the distinctive features between 167 NRL fibers that fold in a prototypical two-start geometry and 197 NRL fibers that were suggested to fold in a one-start fiber. We have shown that both one- and two-start geometries fit the same four-state statistical physics model, with very similar dimensions of the states and transition energies. Although these fitted parameters roughly match structural data of the nucleosome, they do not provide insight into the precise energetics and structural transitions at the microscopic level. Therefore, we adapt a rigid-basepair MC framework to simultaneously include un- and rewrapping of nucleosomal DNA and bending and twisting of linker DNA, as well as (un)stacking of nucleosomes in various fiber geometries. Doing so highlights the well-known mechanical properties of DNA as a major parameter in chromatin fiber folding.

We will use the same step-parameter framework for nucleosomal DNA wrapping and nucleosome stacking as for the basepairs in bare DNA and reproduce force-extension curves for different fiber compositions and folding geometries. Like in most experimental force-extension curves, we did not include linker histones in our simulations. Though the kinetics of unstacking could not be reproduced in our simulations, we obtained fair agreement with the experimental results in the low-force regime in which the fibers are fully folded. This allowed for detailed analysis of the energetics of the linker DNA. Thus, we aim for a quantitative description of the processes during force-induced fiber unfolding and a structural understanding of the loosely organized chromatin fibers before nucleosomes start to unstack and unwrap.

## Materials and Methods

### Experimental force extension data

All experimental procedures and the model that was fitted to the curves were described in detail elsewhere (23) and are summarized in the Supporting Materials and Methods.

### Rigid basepair MC simulations of DNA

We extended the HelixMC package (35) for MC simulations of nucleosome (un)wrapping and chromatin fiber folding. Random step parameters of non-nucleosomal DNA were drawn from a distribution that was previously validated for similar buffer conditions as used in our experiments (35). Basepair replacement was evaluated using a standard Metropolis scheme, which included a work term for the DNA tether. The force was linearly increased from 0.1 to 10 pN in typically 10^5^ steps. Starting configurations of straight DNA interspersed by DNA folded into nucleosomes (see below) were equilibrated in 1000 iterations of all basepairs before ramping the force. For generation of force-extension plots, 250 logarithmically distributed points were stored during the simulations, without averaging. To further mimic the experimental conditions, we added a hard-wall potential for both the surface of the flow cell and the bead when the *z*-coordinate of the basepair was smaller than 0 or larger than that of the last basepair. Hard-wall potentials were set to 10^6^ k_B_T. This simulation scheme resulted in force-extension curves of DNA that closely matched a WLC with a persistence length of 50 nm and without hysteresis, indicating full equilibrium. Though the resulting thermal fluctuations bear close resemblance with experimental curves, part of the fluctuations in the experimental data is filtered out because of the large viscous drag of the bead at small forces.

### Nucleosome (un)wrapping in MC simulations

The wrapping of DNA into nucleosomes was implemented using a second layer of step parameters corresponding to the 14 DNA histone contact points in the nucleosome, as depicted in Fig. 2, *b* and *c*. From the Protein Data Bank (PDB): 1KX5 entry, the average B-factor was computed for all basepairs. 14 fixed basepairs were picked from 14 local minima of the B-factor. Note that this reduces the number of nucleosome-wrapped basepairs to 132 out of 147. Using the frames of these fixed basepairs, 14 step parameters were computed relative to the frame of the dyad basepair. The stiffness matrix for the wrapping parameters has not been described before to our knowledge. We assumed an SD of the step parameters of 1 Ǻ and 0.1 radian to define the diagonal of the stiffness matrix. Outcomes did not change notably for slightly different values. Unwrapping energies were clipped to 2.5 k_B_T to match known histone-DNA contact energies (36), yielding nucleosomes that can unwrap from the entry and exit points of the nucleosome. By defining the histone-DNA contact step parameters relative to the dyad basepair frame, we allowed for full unwrapping, which can occur in an asymmetric fashion. The boundary of nucleosomal DNA shifts into the nucleosome in forward iterations. Once inside the nucleosomal DNA, step parameters were kept unchanged up to the first free basepair on the other side of the nucleosome. If the first free basepair was within the range of nucleosomal basepairs, new step parameters were drawn from the corresponding basepair in the crystal structure rather than from a random pool and were accepted following the standard Metropolis criterion. Thus, rewrapping of nucleosomal DNA was implemented at the end of the nucleosome. This excursion from the standard MC scheme allowed for rewrapping of nucleosomal DNA, which could not be achieved with the default step parameter replacement because of the large difference in step parameters of the nucleosomal DNA with respect to B-DNA. We tuned the wrapping energy such that the unwrapping force of nonstacked nucleosomes matched the experimentally found 2.5 pN. The absence of hysteresis in simulated pull and release curves implies that the condition of detailed balance was met. To impose symmetry in the unwrapping mechanism, the iteration direction of the MC computation was reversed in odd and even iterations. The wrapping energy term was complemented by a hard-wall excluded volume term for all nucleosome pairs with center-to-center distances smaller than 5 nm.

### Nucleosome stacking in MC simulations

Nucleosome stacking was parametrized using a third layer of step parameters that describe the reference frames of the nucleosomes, as shown in Fig. 2 *a*, similar to Korolev et al. (37). However, we only used DNA coordinates to obtain the nucleosome reference frame. We define this frame with its origin at the center of mass of the nucleosome, the *x* axis pointing to the dyad and the *z* axis pointing along the direction of the nucleosomal DNA super helix and the *y* axis perpendicular to the *x* and *z* axis. Nucleosome step parameters were defined by positioning the nucleosomes in a left-handed one- or two-start helix, shown in Fig. 2, *b* and *c*. The dimensions of the chromatin fiber were chosen to roughly match the geometries obtained from condensed fibers using EM: an outer diameter of 33 nm, a nucleosome line density of 2 nm, and seven nucleosomes per super helical gyre (20). Note that these measurements were obtained from fibers containing linker histone H5, which was not included in our simulations. Nevertheless, in our force spectroscopy measurements, we did not observe differences in extension with or without linker histones up to the unstacking transition (21), suggesting a similar packing geometry. Using nucleosomes casted in this manner, nucleosome step parameters were calculated from the nucleosome reference frames of either neighboring nucleosomes, in the case of a one-start fiber, or next-neighboring nucleosomes for two-start fibers. Note that this imposes identical step parameters for nucleosomes stacked in both geometries, reflecting the local similarity in the structure of stacked nucleosome pairs.

The stiffness matrix for nucleosome stacking is unknown. For our simulations, we assumed an SD due to thermal fluctuations of 10 Ǻ and 1 radian to define the diagonals of the shift and rotation parameters in the stiffness matrix. This relatively low stiffness, reflecting the high flexibility of the H4 histone tails that have been indicated to physically mediate nucleosome stacking (38), allows for significant rearrangements of the nucleosomes in the fiber while maintaining a defined global topology. By clipping the maximal energy for stacking fluctuations to 25 k_B_T, unstacking of nucleosome pairs was allowed for sufficiently large forces. Smaller unstacking energies yielded rupturing of stacked nucleosomes at smaller forces. The structure of the fibers up to these rupture events, however, did not depend on the stacking energy.

## Results

### Comparison of experimental unfolding of chromatin fibers with different nucleosome repeat lengths

**Figure 1:**
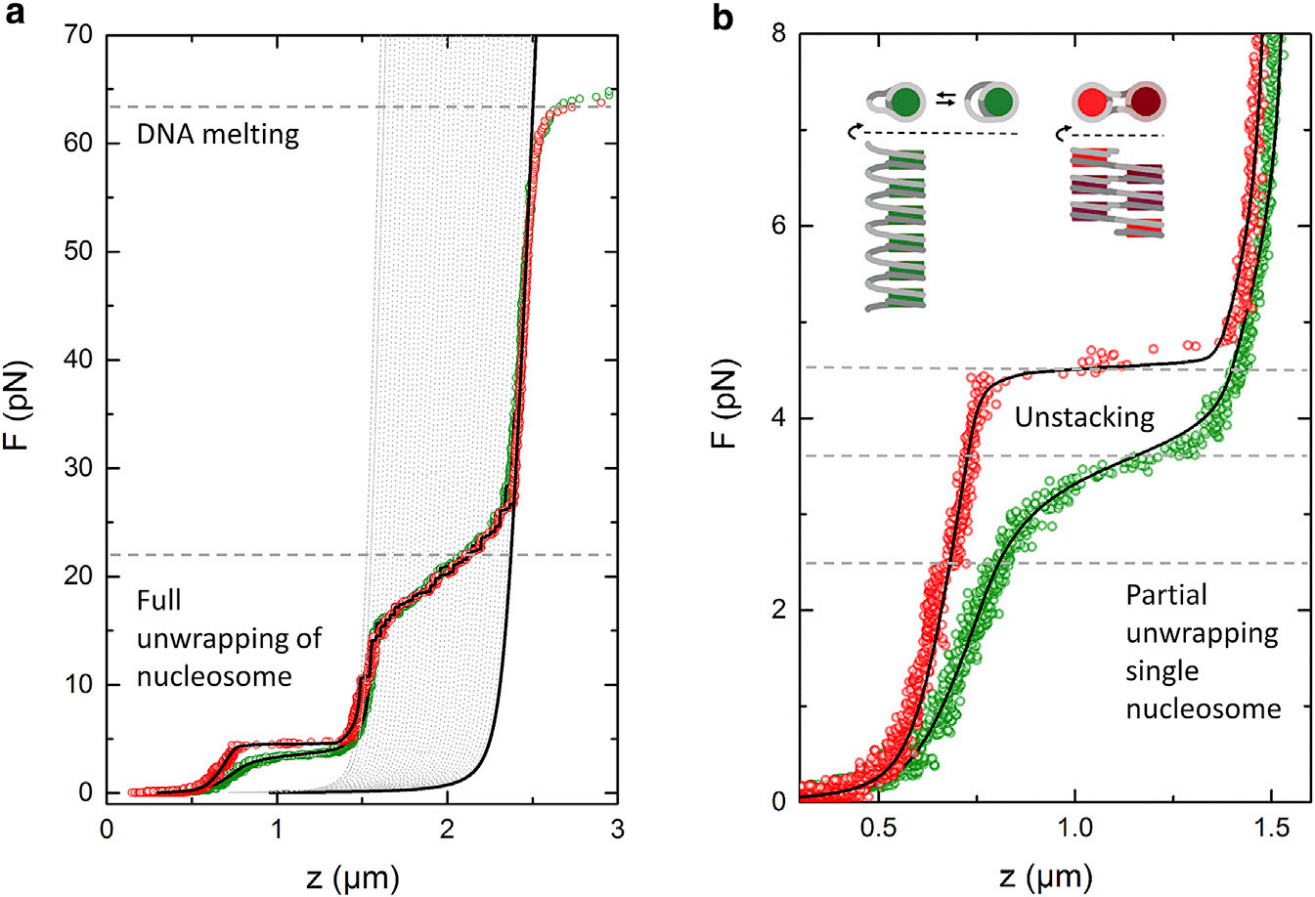
Experimental force-extension curves of individual folded 167 NRL (*red*) and 197 NRL (*green*) chromatin fibers show a difference in stretching and unfolding at forces below 5 pN. (*a*) At 65 pN, the curves exceed the extension of a worm-like chain (WLC) as the DNA overstretches. Multiple 25 nm steps between 10 and 30 pN indicate the unwrapping of the last turn of DNA form each nucleosome. Gray dotted lines correspond to WLC curves that are each 80 bp shorter, corresponding to DNA released from a single nucleosome. (*b*) A zoom-in of the low-force transitions. The 167 NRL fiber unstacks at 4.5 pN in a sharp transition. The 197 NRL fiber unstacks more gradually at 3.5 pN. In addition, a small, 25 nm step is visible in the red curve at 2.5 pN that we attribute to the unwrapping of a single, isolated nucleosome. The inset schematically shows the structural difference between a one-start (*red*) and a two-start (*green*) fiber. Black lines show a fit to a statistical mechanics model that captures the low-force transitions, as described in (23), and a WLC. The fitted parameters and standard errors of the 167 NRL fiber, reconstituted on a 7040 bp DNA substrate containing 30 × 167 repeats of the 601 sequence, were as follows: number of nucleosomes = 30, number of tetrasomes = 3, stiffness = 0.97 ± 0.03 pN/*μ*m, *Δ*G_1_ = 20.0 ± 0.1 k_B_T, and *Δ*G_2_ = 5.7 ± 0.1 k_B_T. The fitted parameters and standard errors of the 197 NRL fiber, reconstituted on a 7125 bp DNA substrate containing 25 × 197 repeats of the 601 sequence, were as follows: number of nucleosomes = 24, number of tetrasomes = 8, stiffness = 0.31 ± 0.01 pN/*μ*m, *Δ*G_1_ = 20.0 ± 0.1 k_B_T, and *Δ*G_2_ = 5.8 ± 0.1 k_B_T. To see this figure in color, go online.

We reconstituted nucleosomes on 7040 bp DNA containing 30 × 167 and 7125 bp DNA with 25 × 197 repeats of the Widom 601 nucleosome positioning sequence to create chromatin fibers with the approximately the same DNA contour length. Fig. 1 shows experimental force-extension curves of a 167 NRL fiber (*red*) and a 197 NRL fiber (*green*). Both curves feature three large transitions in extension: at 65 pN, the DNA overstretches into a 1.6 times longer overstretched structure. At 15–30 pN, the nucleosomes yield a single turn of DNA, visible in 33 distinct 25 nm steps for the 167 NRL fiber and 32 steps for the 197 NRL fiber. Both transitions are similar for 167 and 197 NRL and therefore independent of linker length.

Between 3 and 5 pN, a chromatin overstretching transition occurs that is different for both fibers. The slope of the 167 NRL fiber is almost four times larger, the maximal extension is two times less, and the transition of the 167 NRL fiber is at a higher force and is sharper than that of the 197 NRL fiber. These characteristic differences reproduce our previous reports (21, 23, 34). It is noteworthy to mention that the chromatin overstretching plateau occurs at 1 pN larger force than the unwrapping of a single nucleosome under identical experimental conditions, indicating that the force to rupture interactions between the nucleosomes exceeds that of the unwrapping of DNA around the histone core in single nucleosomes (39). In fact, close inspection of the 167 NRL curve in Fig. 1 *b* reveals a small step in extension at 2.5 pN, suggesting that a single nucleosome partially unwrapped at this force. The detailed difference in structural transitions at low force reflect the (un)folding of the chromatin fibers and suggest a different geometry of the two fibers before unfolding.

A statistical physics model that we developed previously (23), in which we distinguish four states of chromatin folding, was fitted to the experimental data and is represented by the black lines in Fig. 1, *a* and *b*. With increasing force, the nucleosomes undergo a transition from a stacked state via a partially unwrapped state and a single-turn wrapped state to a fully unwrapped state (23). Transitions between each of the states are accompanied by a free-energy cost, which is compensated by the additional work that is done through the increased extension of each subsequently unfolded state. The stiffness and unstacking energy per nucleosome were independent of the number of nucleosomes in the fiber (see Fig. S1). More details and the observation that small compositional differences between fibers can dominate the force-extension curve can be found elsewhere (23). Here, we want to stress that all fitted parameters are highly reproducible between curves and can be summarized in the above-described trends.

The higher stiffness, smaller maximal extension, and cooperative unfolding of the 167 NRL fiber are all consistent with having two stacks of nucleosomes, like in the two-start fiber. This can easily be explained in terms of a simple toy model. When the connecting linker DNA is ignored, it is clear that splitting a single stack of nucleosomes into two stacks of half the number of nucleosomes quadruples the stiffness and halves the maximal extension. As the force would be divided between the two parallel stacks, each stacked nucleosome pair would be exposed to less force and thus yield a higher rupture force. The sharp overstretching transition can be understood when including the linker DNA in the two-start helix. In that case, the internal nucleosomes are further stabilized by their neighbors in the other stack, whereas the end nucleosomes miss such stabilization, resulting in a cooperative rupturing of the fiber that starts with the end nucleosomes. The absence of cooperativity in 197 NRL fibers is therefore another indication of a one-start helix. We recently substantiated this interpretation by covalently linking the H4 tails to the acidic patch of their stacking partner, which yielded an increased rupture force but unchanged stiffnesses for both types of fibers as compared to the non-cross-linked fibers (34). Thus, single-molecule force spectroscopy reveals qualitative and quantitative differences in stretching and unfolding of the higher-order structures of different NRLs that can be rationalized by an altered stacking order of the nucleosomes.

Though the DNA topology in 167 and 197 NRL fibers may be different, the local stacking interaction between two interacting nucleosomes should be similar. From the extension of the fibers at low forces, it is clear that the stacking of nucleosomes in a folded fiber cannot be closely packed such as in nucleosome crystals (15). We measure a maximal extension per nucleosome of 10 nm, which is significantly more than the 6 nm height of the globular part of the nucleosome (40). This gap can just be spanned by the H4 histone tails that mediate nucleosome stacking. In the high-resolution cryoEM (cryogenic electron microcopy) structure (16) and recent force spectroscopy data at very low salt (41), nucleosomes appear to be organized in tetramers, with pairs of more closely packed nucleosomes alternating with pairs of nucleosomes featuring larger gaps. Under more physiological conditions, recent single-pair Förster resonance energy transfer measurements suggest highly dynamic organization in which stacking pairs may rapidly change (42). Nevertheless, we expect that nucleosome stacking, mediated by very flexible, unstructured histone tails that transiently interact, is locally very similar in the two geometries. This implies that linker DNA largely defines the global structure of the chromatin fiber.

### Extension of a rigid basepair model with nucleosomes

**Figure 2:**
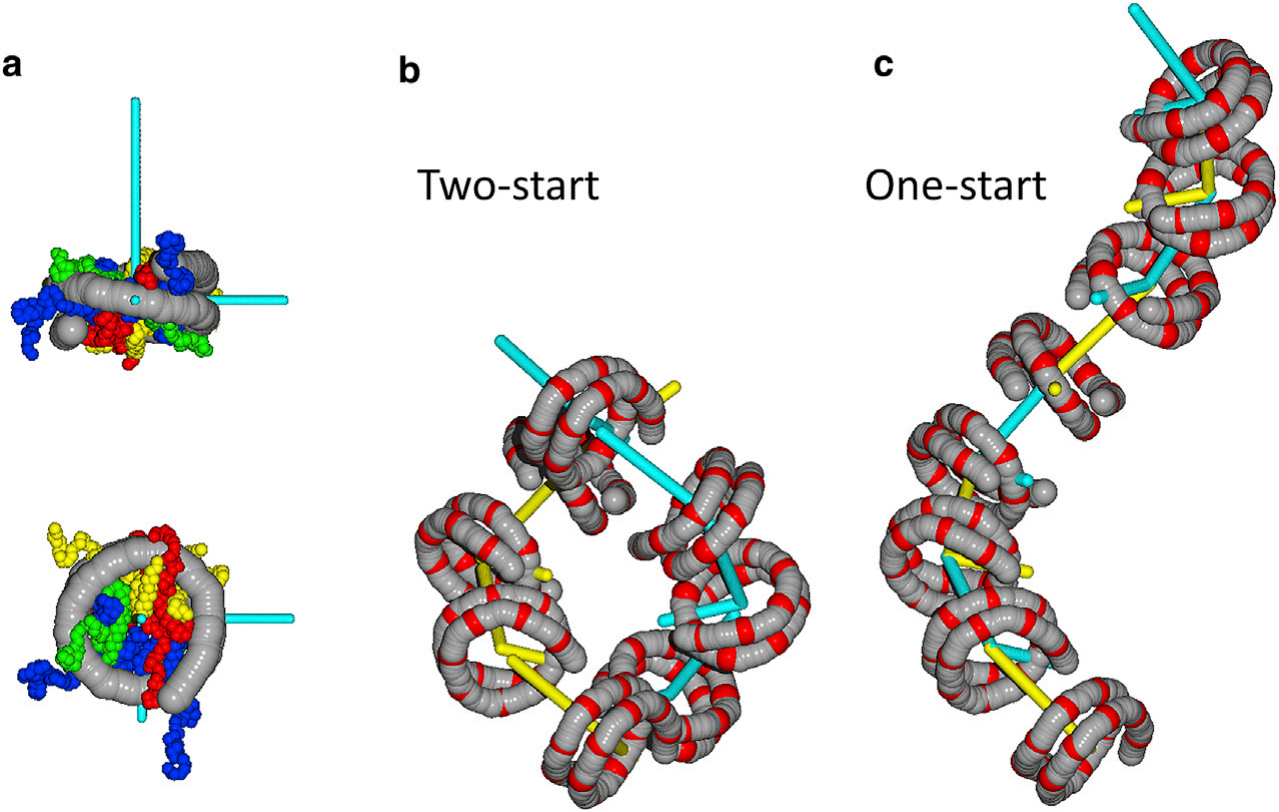
Two additional layers of step parameters define the folded chromatin fiber. (*a*) A nucleosome frame (*cyan*) was created based on the coordinates of the 14 bp that are fixed in the nucleosome (colored *red* in (*b*) and (*c*)), as determined from the local minima of the average B-factors of the DNA coordinates in nucleosome structure 1KX5 (40). The center of mass of the central eight fixed bp, forms the origin of the frame. The *x* axis points to the dyad, defined by the bp that is halfway between the central two fixed bp. The *z* axis is parallel to the vector connecting the center of mass of the first seven fixed bp with the center of mass of the last seven fixed bp. (*b*) A two-start helix is constructed by arranging odd nucleosomes (*yellow frames*) and even nucleosomes (*cyan frames*) in a helical structure. The *y*-vectors of the nucleosome frames were omitted for clarity. (*c*) Stacking odd nucleosomes on even nucleosomes defines a one-start helix. Note that the relative orientation of the nucleosomes is highly similar in both structures and the parametrization of the six kinematic parameters of these structures is used to impose fiber folding in the MC simulations. To see this figure in color, go online.

To model the linker DNA in folded chromatin fibers, we extended the HelixMC package (35) to include nucleosomal DNA wrapping and nucleosome stacking. The HelixMC package was based on the work of Olson and co-workers, who captured the mechanical properties of DNA in six sequence-dependent kinematic parameters (43). Here, we include two extra layers of kinematic parameters that define DNA wrapping around the histone core and stacking of nucleosomes in the fiber. We used the DNA step parameters of the 1KX5 crystal structure of the nucleosome (40) to replace the step parameters in a random pose of DNA. The frames of 14 basepairs with the lowest B-factor were each assigned an energy of up to 2.5 k_B_T to account for deviations from the crystal structure; see Fig. 2.

**Figure 3:**
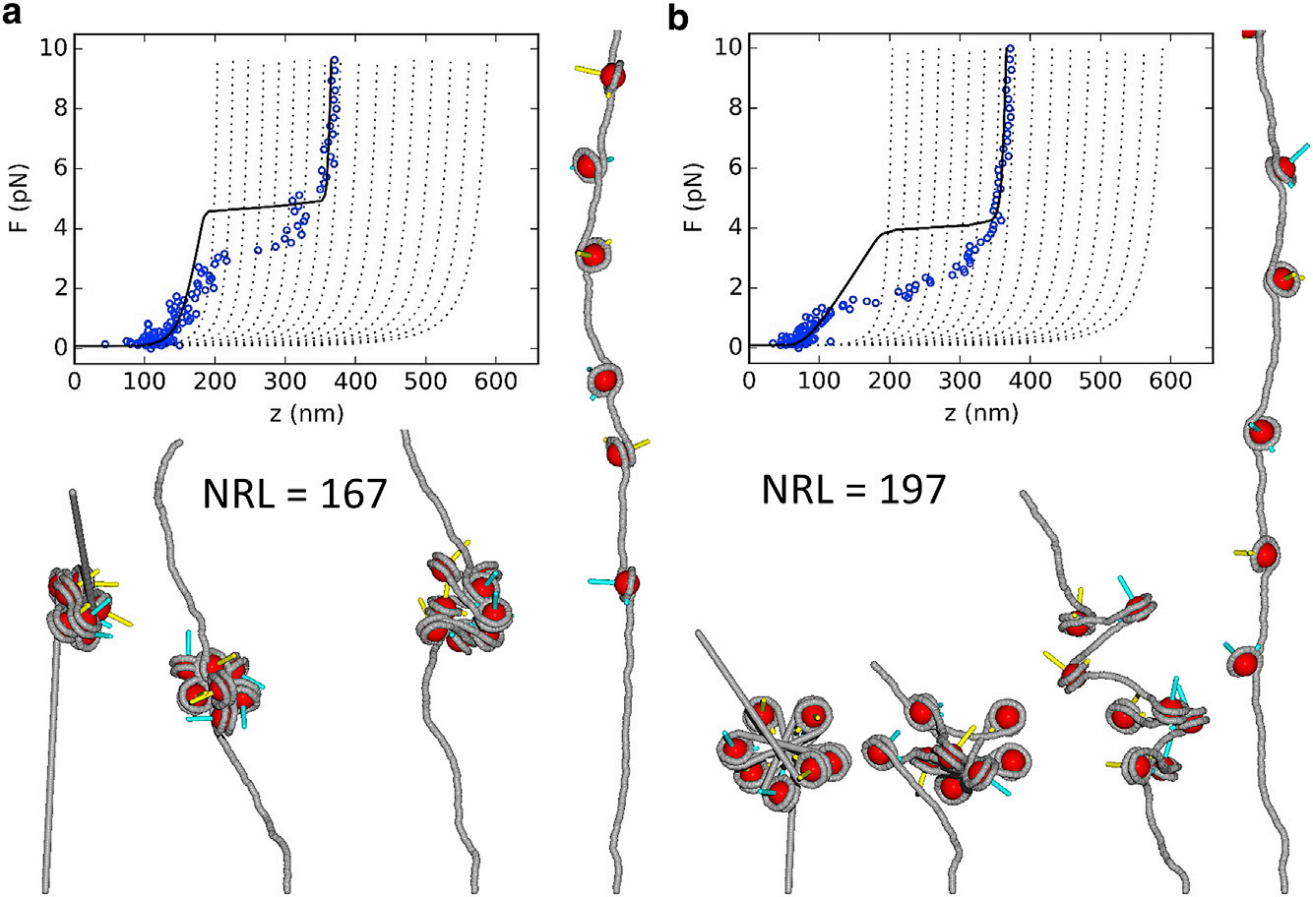
MC simulations of arrays of noninteracting nucleosomes that readily unfold when the force is increased up to 10 pN. (*a*) A simulated force-extension curve of an 1800 bp 167 NRL fiber containing eight nucleosomes (Video S1). (*b*) A simulated force-extension curve of an 1800 bp 167 NRL fiber containing eight nucleosomes (Video S2). The nucleosomes release their first turn of DNA at 2.5 pN. Black lines show the statistical mechanics model for fiber unfolding using parameters fitted to experimental curves for both fibers. Gray dotted lines are plotted for reference and represent WLC curves in which the contour length is increased for both unwrapping events of each nucleosome. The snapshots below the curve show the structures of the initial conformation (*left*) and conformations at 0.1, 1, and 10 pN. Note that the top part of the tallest structures is clipped from the figure. To see this figure in color, go online.

Fig. 3 shows the results of MC simulations of nucleosomal arrays consisting of eight noninteracting nucleosomes reconstituted on an 1800 bp DNA substrate. In 50,000 steps, the force was ramped linearly from 0.1 to 10 pN and back. In 1000 preceding steps, the nucleosomes were forced into a stacked conformation (for proper comparison with other simulations, see below), but because there is no nucleosome-nucleosome interaction potential in the remainder of the simulation, the nucleosomes behave largely independently in the remaining trajectory. These initial 1000 steps were discarded. Snapshots of the initial structure up to the structure at 10 pN are shown below the force extension curve. Videos of the structures corresponding to all traces are available as Supporting Material. Both force-extension curves feature an unwrapping transition at 2.5–3 pN and converge to a worm-like chain (WLC) with a contour length of 1800 − 8 × 80 = 1160 bp, corresponding to a single wrap of DNA around each nucleosome, in close agreement with experimental data (39). Interestingly, the nucleosomes in the 167 NRL array unwrap at higher forces than those in the 197 NRL array, probably because unwrapping is somewhat held up by steric hindrance of the nearby nucleosomes.

Video S1. Rigid Basepair MC Simulation of an 1800 bp 167 NRL fiber Containing Eight Noninteracting Nucleosomes

Video S2. Rigid Basepair MC Simulation of an 1800 bp 197 NRL Fiber Containing Eight Noninteracting Nucleosomes

Overall, these simulations largely reproduce the experimental behavior of eight times a single nucleosome, which features the unwrapping of the first turn of DNA at 2.5 pN (39). The increased condensation of the 8 × 197 NRL fiber at low forces relative to a WLC with a contour length that is reduced by 8 × 147 bp should be attributed to the kink in the DNA trajectory that is induced by the nucleosomes (44). Strikingly, at forces below 1 pN, the simulated force-extension curves overlap with the statistical physics model based on the experimental data—which are plotted in black—suggesting that this level of condensation can be achieved without attractive nucleosome-nucleosome interactions. However, the significantly higher level of the overstretching plateau in the experimental data suggests that we need nucleosome-nucleosome stacking interactions to obtain a better match.

### Nucleosome stacking in two-start fibers

**Figure 4:**
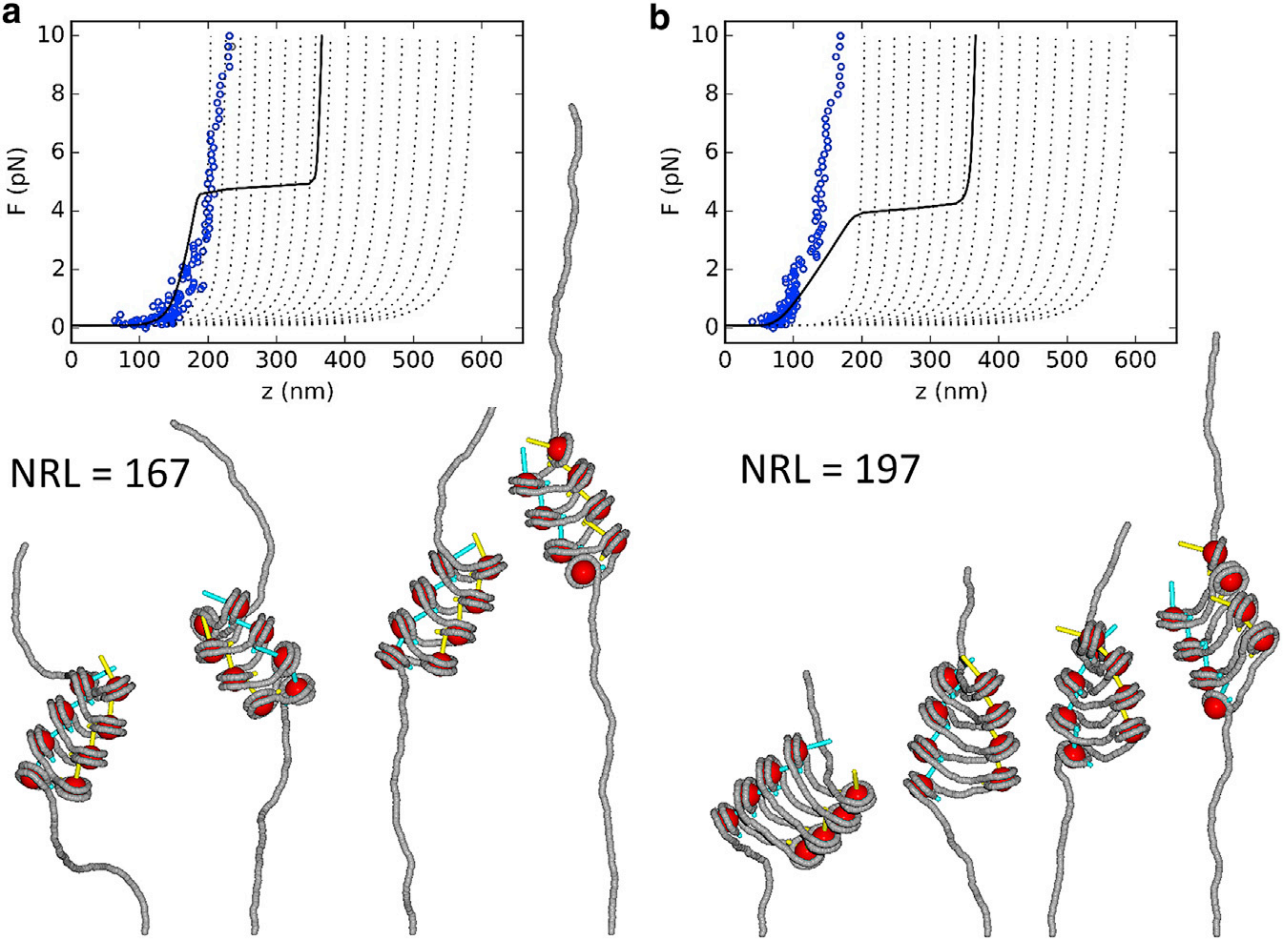
MC simulations of the stretching of two-start fibers show a high stiffness. (*a*) A simulated force-extension curve of a two-start 167 NRL fiber containing eight nucleosomes (Video S3). (*b*) The force-extension curve of a two-start 197 NRL fiber (Video S4). The snapshots below the curves show the structures of the fiber at 0.1, 1, 2, and 10 pN. To see this figure in color, go online.

The linker DNA in two-start fibers does not bend severely to accommodate nucleosome stacking. The structures in Fig. 4 show nevertheless that a balance between optimal stacking and minimizing the bending of the linker DNA is found in the simulations. Optimal stacking would place the ends of the colored nucleosome frame *z*-vectors in the center of the next nucleosome, which is clearly not the case for both fibers. In the simulations, we do not observe the typical overstretching transition at 3–5 pN, even when the force is increased up to 10 pN. The stacked nucleosomes appear kinetically trapped because we estimate that the work associated with unwrapping and unstacking at 10 pN is about twice the used stacking energy. Only the outer two nucleosomes feature DNA unwrapping, but the stacking is maintained throughout fibers. This is consistent with a cooperative transition for overstretching the two-start fibers. However, because detailed balance was only achieved when nucleosomes remain stacked, we refrain from a kinetic interpretation at higher forces.

Video S3. Rigid Basepair MC Simulation of an 1800 bp 167 NRL Fiber Containing Eight Nucleosomes Folded in a Two-Start Fiber

Video S4. Rigid Basepair MC Simulation of an 1800 bp 197 NRL Fiber Containing Eight Nucleosomes Folded in a Two-Start Fiber

The extension of the fibers agrees with the experimental extension at low forces but deviates at forces exceeding 1 pN. In the 167 NRL fiber, a larger extension is found in the simulations that is caused by the partial unwrapping of the outer nucleosomes as depicted in the snapshots below the curve. It never exceeds the equivalent of 60 bp of unwrapped DNA. Partial unwrapping of the outer nucleosomes also occurs in the 197 NRL fiber, but this is not sufficient to reach the experimentally observed extensions for this type of fiber. Some extra extension, corresponding to roughly the linker length, is gained at higher forces by shearing the two stacks of nucleosome. Both the unwrapping at the ends and the shearing of the stacks do not scale with the number of nucleosomes, though, so we expect that these effects become negligible in simulations of larger arrays of nucleosome as used in the experiments. The simulated force-extension curve of a two-start 167 NRL fiber would overlap with the experimental curves up to the overstretching plateau, when end effects are corrected for, but the stiffness of the simulated two-start 197 NRL fiber is significantly larger than measured experimentally, suggesting that this fiber folds in a different manner.

### Nucleosome stacking in one-start fibers

**Figure 5:**
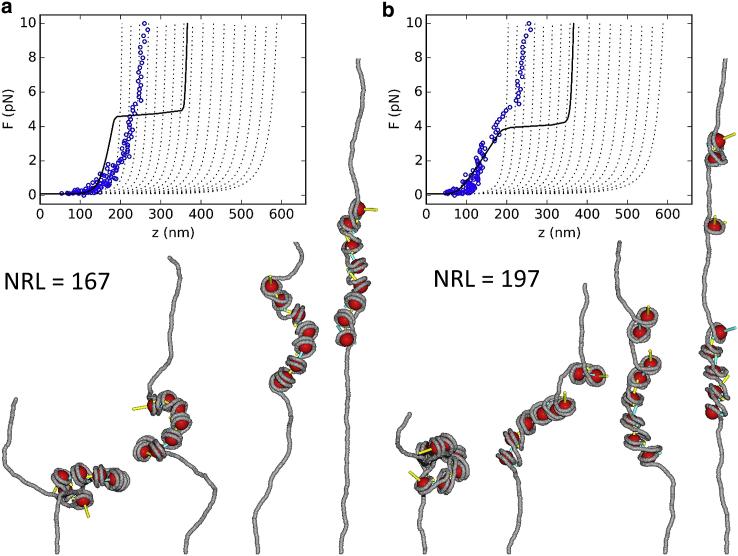
MC simulations of the stretching of one-start fibers feature a lower stiffness. (*a*) A simulated force-extension curve of the one-start 167 NRL fiber containing eight nucleosomes (Video S5). (*b*) The force-extension curve of a one-start 197 NRL fiber (Video S6). The snapshots below the curves show the structures of the fiber at 0.1, 1, 2, and 10 pN. To see this figure in color, go online.

The force-extension curves in Fig. 5 started with fiber structures that were forced into a one-start helix before ramping up the force. Although the linker DNA features big curvatures, especially in the case of the 167 NRL fiber, the nucleosomes largely remain trapped in the stacked state over the entire force range. In both cases, the stacking seems to be better optimized than for the two-start fibers because the *z*-vectors of the nucleosome frames are much closer to the centers of the next nucleosomes. At larger forces, the stacking gets compromised because the helical stack of nucleosomes is extended. The end nucleosomes were prone to partial unwrapping, like in the two-start structures. In the 197 NRL fiber, we observed a rare event of a stacking defect at ∼1 pN, which leads to further unwrapping and the release of extensive lengths of linker DNA at larger forces. Overall, one-start 197 NRL fibers were more fragile than one-start 167 NRL fibers despite the larger bending stress in the latter. The larger work associated with the unstacking in 197 NRL fibers makes them more susceptible to force.

Video S5. Rigid Basepair MC Simulation of an 1800 bp 167 NRL Fiber Containing Eight Nucleosomes Folded in a One-Start Fiber

Video S6. Rigid Basepair MC Simulation of an 1800 bp 197 NRL Fiber Containing Eight Nucleosomes Folded in a One-Start Fiber

Compared to the experimental model, the one-start 167 NRL fiber was more extended at forces larger than 1 pN. For the one-start 197 NRL fiber, there is a better match. However, the stiffness of the simulated fiber exceeded that of the experimental model. Though the simulations do not perfectly capture the force-extension behavior in this case, the stiffness is roughly four times smaller than that of the two-start fiber. Further relaxing of the stacking step parameters may yield a better agreement with the experimental data, but the obtained ratio of stiffnesses confirms the toy-model comparison of one-start versus two-start stretching.

### MC simulations of larger chromatin fibers

**Figure 6:**
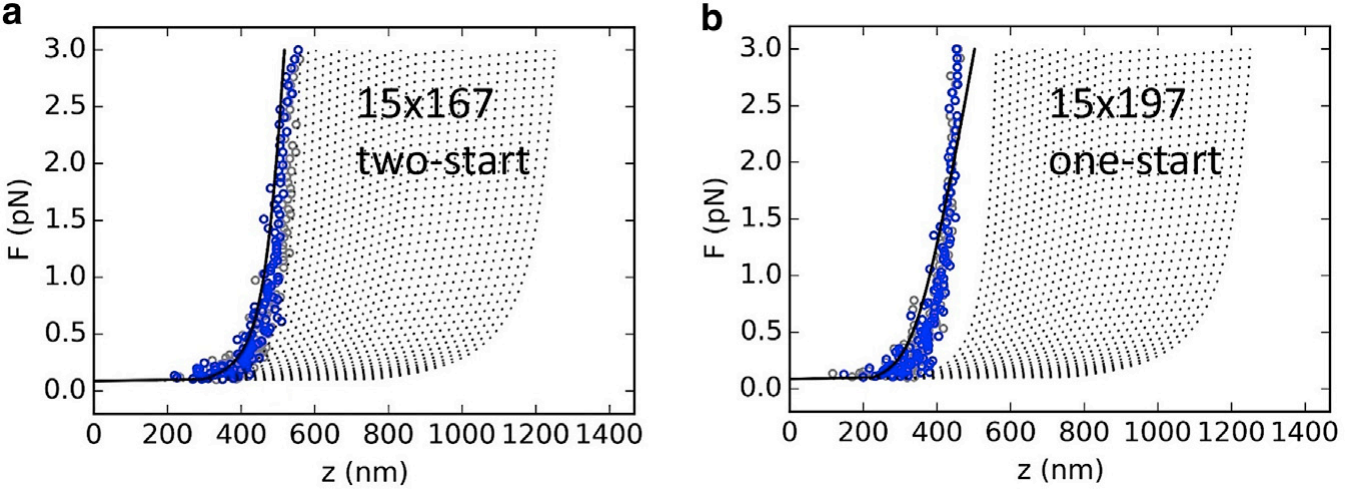
MC simulations of 4000 bp fibers containing 15 nucleosomes reproduce experimental data up to the unstacking transition. (*a*) A 167 NRL fiber in a two-start conformation. (*b*) A 197 NRL fiber in a one-start conformation. Blue dots represent pulling curves, gray dots the subsequent release curve, and the black line the statistical physics model with a stiffness corresponding to the stiffness that was fitted to the experimental data. To see this figure in color, go online.

To better compare the MC simulations with our experimental data, we simulated fibers containing 15 nucleosomes at forces increasing up to 3 pN and subsequent release back to 0.1 pN. Because this is well below the chromatin-fiber overstretching transition that we attributed to nucleosome unstacking, the corresponding large increase in extension was fully absent, as shown in Fig. 6. The extension of the 167 NRL two-start simulated data (Fig. 6 *a*) showed over the entire force range good agreement with the modeled curve, plotted in black. The 197 NRL fiber was more condensed (Fig. 6 *b*), both in the simulations and in the model curve. However, the simulated data featured a higher stiffness than the model based on experimental data.

Whereas the extension of the 167 NRL fiber was slightly less than the extension that can be expected for noninteracting nucleosomes, the 197 fiber was more than 100 nm shorter over most of the force range. At very low forces, however, the difference is negligible. In a separate simulation, we tracked the extension of these fibers in absence of force (data not shown). We obtained extensions *z* = 0.29 ± 0.09, 0.28 ± 0.09, and 0.26 ± 0.09 *μ*m (mean ± SD) for 167 NRL without stacking, 167 NRL fibers in a two-start structure, and 197 NRL fibers in a one-start structure. Note that the extension of the fibers was dominated in this case by the large DNA handles (1535 and 1145 bp for 15 × 167 NRL and 15 × 197 NRL DNA substrates), similar to DNA substrates used in the experiments.

The overlap of the pull and release simulations shows that the MC simulation is fully reversible. The condition of detailed balance was corroborated by analysis of the autocorrelation of the extension and the unwrapping energy at zero force, shown in Fig. S2. Correlation in the extension could be observed up to 30 steps in all fiber conformations. The unwrapping was somewhat slower, with vanishing correlations after ∼200 MC steps for stacked nucleosomes and ∼75 MC steps for noninteracting nucleosomes.

Like in the shorter fibers, we observed only a small amount of unwrapping in the 15 nucleosome fibers. In absence of force, the unwrapping energy per nucleosome was 0.7 ± 0.5, 0.6 ± 0.3, and 0.3 ± 0.3 k_B_T (mean ± SD) for 167 NRL without stacking, 167 NRL in a two-start structure, and 197 NRL in a one-start structure, respectively. The energy penalty for the release of 10 bp of DNA was set at 2.5 k_B_T, so at any time in the simulation, only roughly a quarter of the nucleosomes featured the release of one out of 14 histone DNA interactions. The small variations in unwrapping energy confirmed the absence of significant transient unwrapping. Thus, though our model only imposed wrapping of 132 out of 147 bp of the nucleosome, further unwrapping of nucleosomal DNA did not significantly contribute to stabilization of the stacking interactions.

### Evaluation of the energies associated with fiber folding

The MC simulations not only result in three-dimensional structures of the chromatin fibers with force-extension curves that compare well with the experimental data, they also allow for a detailed comparison of the energies associated with the dynamic structures. In Table 1, we summarized the energy contributions of the folded fibers relative to fibers with the same NRL but without stacking interactions. To cancel the large variations between static structures, we averaged the data between 0.1 and 1.5 pN, in which force range all nucleosomes were stably stacked. We calculated the energies from the step parameters and the stiffness matrices. To exclude end effects, we only considered DNA sections from dyad to dyad.

**Table 1:** Quantification of the Energy Changes upon Stacking of Nucleosomes in One- and Two-Start Fibers Shows that Nucleosomes in Two-Start 167 NRL Fibers and One-Start 197 NRL Fibers Can Both Reduce Their Energy with ∼8 k_B_T When Nucleosomes Stack

NRL	167	167	197	197
Fiber	One-Start	Two-Start	One-Start	Two-Start
Shift	0.5 ± 0.4	−0.3 ± 0.3	−0.7 ± 0.3	−0.6 ± 0.4
Slide	1.3 ± 0.4	0.1 ± 0.2	0.3 ± 0.1	0.4 ± 0.5
Rise	1.6 ± 0.3	0.0 ± 0.4	−0.7 ± 0.4	−0.4 ± 0.7
Tilt	4.9 ± 0.6	1.3 ± 0.2	3.8 ± 0.7	−1.8 ± 0.7
Roll	10.3 ± 0.1	2.5 ± 0.7	8.7 ± 0.6	−1.3 ± 1.0
Twist	6.1 ± 0.8	−0.6 ± 0.2	1.0 ± 1.2	−1.6 ± 1.6
Σ step	24.7 ± 2.3	3.1 ± 0.5	12.4 ± 1.8	−5.3 ± 2.3
Wrap	0.6 ± 0.4	−0.3 ± 0.1	−0.4 ± 0.2	−0.6 ± 0.1
Stack	−22.7 ± 0.2	−11.3 ± 0.5	−19.8 ± 1.7	−11.8 ± 0.4
Total	2.6 ± 2.8	−8.6 ± 0.7	−7.8 ± 0.4	−17.6 ± 2.0

For comparison, the average bp step energies were calculated between F = 0.1 and 1.5 pN and summed between pairs of neighboring dyads. The corresponding energies of the nonstacking fibers (shown in Fig. 3) were subtracted. All energies are expressed in units of k_B_T. Errors represent SDs obtained from four separate simulations. Σstep is the sum of the energies that correspond the six degrees of freedom of the bp and quantifies the penalty for linker DNA bending and twisting. Together with the wrapping and the stacking energy, we calculated the total change in energy upon nucleosome stacking in this framework.

As expected, the one-start fibers feature a large energetic penalty for bending the linker DNA, which is exemplified in the energy differences defined by roll of the basepairs: 10.3 and 8.7 k_B_T for 167 and 197 NRL fibers. The one-start 167 NRL fiber also has excessive energies for tilt and twist. For the two-start 167 NRL fiber, it appears that excluded volume effects and unfavorable twisting of the linker DNA to accommodate stacking do not favor linker conformations relative to those of nonstacking nucleosomes. Only in the two-start 197 NRL fiber is the energy of the linker DNA reduced as compared to nonstacking nucleosomes.

Previously, we suggested that partial unwrapping of the nucleosomal DNA may relieve some of the bending of the linker DNA in one-start fibers (23). In these simulations, it appears that this is not the case: the averaged energy associated with unwrapping is within 1 k_B_T for all fibers. Note, however, that we fixed the histone-DNA contacts in the nucleosome at 14 frames, which constrains only 132 bp. The outer 15 bp were therefore free to move, and this allowed for substantial reduction of the bending of the linker DNA. The small values and fluctuations of the wrapping energy, relative to 2.5 k_B_T per contact point, indicate that the central 132 bp of nucleosomal DNA remain firmly wrapped around the histone core at low forces.

Surprisingly, we find the largest variation in the stacking energy. One-start fibers appear to be much better positioned to optimize stacking between nucleosomes than two-start fibers. This is not caused by steric hindrance of the linker DNA because both structures can fold in the same relative orientation of stacked pairs of nucleosomes without clashes of the linker DNA. It rather seems that the linker DNA of a pair of nucleosomes in a two-start conformation positions the nucleosome in between, in an unfavorable orientation for stacking with its next neighbor. Excluded volume effects between the nucleosomes, which were included in the MC simulations, may complement to this effect. Though stacking is better in one-start fibers, none of the fiber geometries position the nucleosomes in a perfect orientation, yielding a realized stacking energy that is reduced 3–13 k_B_T less than the set stacking energy.

When all energy contributions are summed, two-start 167 NRL fibers and one-start 197 NRL fibers are both ∼8 k_B_T per nucleosome lower in energy than fibers consisting of noninteracting nucleosomes. The two-start 197 NRL fiber appears to be most stable, reducing the energy another 10 k_B_T. The stacking energies are well in range with the energy that we fitted for the first transition in the force-extension curve, which are typically 15–19 k_B_T (23). The current simulations, however, do not include entropy calculations and therefore miss an important part of the free energy that would be required for a full comparison. Moreover, the simple harmonic stacking potential may not properly weigh nonoptimal stacking of nucleosomes. This may bias the stacking energy toward two-start conformations. It is clear, though, that each fiber configuration needs to balance optimal nucleosome stacking with minimal linker DNA deformation and that small changes in linker length have a large impact on the energies and the resulting fiber structures.

## Discussion

Single-molecule force spectroscopy of folded chromatin fibers provides a unique way to probe chromatin conformations and dynamics under physiological conditions. Though the extensions of single fibers can be measured with nanometer accuracy and manipulated with pN precision, the resulting force-extension curves cannot unequivocally discriminate between different folding topologies. In this work, we presented a framework of rigid basepair MC simulations supplemented by nucleosome wrapping and nucleosome stacking step parameters that provides quantitative insight into the energy contributions that define folded chromatin fibers. We simulated two frequently studied NRLs and evaluated the force-extension relation and the energy contributions of three different nucleosome stacking options. The results largely agree with previous interpretations of the force-extension curves and uniquely quantify and visualize the delicate balance between optimal nucleosome stacking and minimal deformation of the linker DNA. The simulations disclose the very dynamic structure of the fibers that can best be seen in the videos (see the Supporting Material). The relatively disordered structures, despite strong stacking interactions and the stiff and stressed linker DNA, may be inhibitive for experimental techniques that rely on averaging, which would partially explain reported absence of chromatin higher-order structure (45).

The novelty of our simulations, to our knowledge, lies in the quantification of the energy required for shaping the linker DNA to accommodate fiber folding by nucleosome stacking. The rigid-basepair model has been shown to successfully reproduce detailed mechanical features of DNA. Our work and that of Norouzi and Zhurkin (33) report on the effects of constraints set by interacting nucleosomes in folded fibers. Whereas Norouzi and Zhurkin (33) fixate the position of pairs of nucleosomes and subsequently evaluate the linker DNA deformation, we allow for some flexibility in the nucleosome positions, as set by the stiffness matrix. Because the stiffness matrix was derived from the global dimensions of folded chromatin fibers, as measured by EM and force spectroscopy, this approach makes the origin of the constraints for nucleosome positions explicit. In combination with optional unwrapping of nucleosomal DNA, it may also better represent the flexible and relatively disordered nature of nucleosome-nucleosome interactions. Moreover, we used the same stacking parameters, which agree with a universal nucleosome-nucleosome interaction independent of the topology of the fiber, in one-start and two-start conformations. This makes it possible to compare the effects of changes in linker DNA length in great detail.

For 167 NRL fibers, both the force-extension curves and energy calculations favor the well-known two-start conformation. This NRL appears to be optimal for a two-start helix because the energy required for twisting the linker DNA is only 0.6 k_B_T and can hardly be reduced. Addition of a single bp will increase the twist by 36° and thus increase the twist energy, which may prevent stacking of nucleosomes in larger NRLs. Future simulations and experiments will need to confirm this hypothesis. Despite the ideal linker length, the stacking is far from optimal. The simulations did not yield the tetranuclesomal patches that were observed in a crystal structure (15), EM reconstruction (16), force spectroscopy (41), and Förster resonance energy transfer experiments (42). Such an arrangement would provide an alternative compromise for balancing linker bending and nucleosome stacking. Nevertheless, the quantitative agreement with experimental force-extension data shows that fibers can be stretched up to 5 nm per nucleosome while remaining fully stacked and wrapped.

In experiments, the 197 NRL fibers yield larger extensions before overstretching, and this trend is only reproduced in the case of the one-start helix. Though the simulations do not fully catch the experimentally obtained large extension and low stiffness, it is clear that the one-start conformation is a better match than the two-start conformation, which cannot reach such large extensions. Nevertheless, the two-start conformation yields the lowest energy for 197 NRL fibers because the linker DNA is relatively straight. However, the experimentally observed extensions can only be achieved when nucleosomes unstack, which costs more energy than the alternative scenario in which a one-start is formed with the same extension. Contrary to popular belief, the energy calculations show that the one-start helix does not present a prohibitively large penalty for linker DNA bending. The ability to find a better stacking conformation largely compensates the 12.4 k_B_T that is required for bending the linker DNA. This may make the one-start helix the preferred structure for 197 NRL fibers. The simulations show that the experimentally obtained force-extension curves of 167 NRL fibers can be fully reproduced by stretching stacked nucleosomes in a two-start conformation. The experimental force-extension curves of 197 NRL fibers give a better, though not perfect, match with a one-start conformation. Yet, the values of the stacking energy of fibers with this repeat length appear to favor a two-start conformation. We attribute this discrepancy to the simplified calculation of the stacking energy, for which we used a harmonic potential. A potential that would penalize large deviations more strongly may rectify this. Indeed, when stacking is mediated by the H4 tails, one would expect a nonharmonic potential. Nevertheless, despite the simplified interaction potential, the simulations show that the force-extension curves of both fibers can be largely reproduced up to the overstretching transition without breaking nucleosome-nucleosome interactions.

The step parameter approach appears to work well for nucleosome (un)wrapping. The energy in each histone-DNA contact point is similar as reported before (36), and the force at which the first unwrapping transition occurs agrees with the experimentally observed force (39). The unwrapping is reversible, and simulated force-extension curves do not show hysteresis, indicating thermodynamic equilibrium. Like in experiments, the second unwrapping transition is not in equilibrium, but the force at which this transition occurs is much higher in the simulations than in the experiments (data not shown). Likewise, the unstacking transition is only rarely observed in the simulations up to 10 pN. Longer simulations may catch the dynamics of the force-extension curves better, but more extensive simulations need to be performed to test whether these transitions can be accurately captured in this MC model or whether more efficient sampling strategies are required. Note that the timescale of MC simulations cannot be coupled directly to the experimental pulling rates.

We assumed that the linker DNA is free from interactions with proteins, which allows for straightforward use of the rigid-basepair model to evaluate linker DNA deformation. The presence of linker histones would change this situation and sets additional geometrical constraints to the linker DNA that go beyond the scope of this work. The current simulations do, however, nicely match the experiments in which we cross-linked the H4 tail to the acidic patch on the neighboring nucleosomes (34), showcasing their relevance for interpretation of the experimental data. Optimization of the stiffness matrix that defines the stacking interactions may further improve the simulations. In the absence of experimental input, we postulated a simple stiffness matrix with only diagonal terms. The used stiffness values form a compromise between high flexibility and imposing only very local interactions. The decisive role of the H4 tails in nucleosome stacking suggests that the interactions may be better modeled by a potential that describes the extension of a freely jointed chain. Other, more refined models, including all-atom simulations (27), can of course provide much more detailed insight. Such models will be able to include the role of electrostatics, histone tails, PTMs, and other essential ingredients of chromatin biology. However, because the local geometry between two stacked nucleosomes is the same in all stacking conformations that we simulated, our approach may yield a fair comparison in which those more local effects remain the same between different geometries. Moreover, coarse-grained structures as presented here may be the input for more refined models.

Awaiting such larger computational efforts, we aim to make the discussion about chromatin fiber structure and the interpretation of force spectroscopy experiments on these fibers more quantitative with our simulations. The highly dynamic features of the chromatin fibers advocate a shift in the paradigm of chromatin structure: rather than looking for perfectly regular, ordered fibers or fully disordered structures, it may be more constructive to describe folded chromatin fibers in terms of interacting pairs of nucleosomes. Although the interactions can be stable, maintaining a fixed topology, the structure will still have enough freedom of movement for relatively large shape changes while locally allowing for transient defects. Such an open and dynamic but well-organized structure may offer the eukaryotic cell the means to organize its genome in an efficient manner and offers many possibilities for further regulation.

## Author Contributions

Single-molecule experiments were performed and analyzed by A.K.; MC simulations were done by B.E.d.J., T.B.B., B.V., and J.v.N.; J.v.N. wrote the manuscript. All authors read and approved the manuscript.

## Supporting Material

Document S1. Supporting Materials and Methods, Figs. S1 and S2, and Tables S1–S3

Document S2. Article plus Supporting Material
