# Supplementary material for: Rigid Basepair Monte Carlo Simulations of One-Start and Two-Start Chromatin Fiber Unfolding by Force
Source: Biophys J. 2018 Oct 11;115(10):1848–59. doi: 10.1016/j.bpj.2018.10.007 (PMC6303278; doi:10.1016/j.bpj.2018.10.007)
Supplement: Document S1. Supporting Materials and Methods, Figs. S1 and S2, and Tables S1–S3 [file mmc1.pdf]

Biophysical Journal, Volume *115*

Supplemental Information

Rigid Basepair Monte Carlo Simulations of One-Start and Two-Start Chromatin Fiber Unfolding by Force

Babette E. de Jong, Thomas B. Brouwer, Artur Kaczmarczyk, Bert Visscher, and John van Noort

Methods

Reconstitution of chromatin fibers. XL-1-blue E.coli competent cells (Agilent Technologies) were transfected with Ampicillin-resistant pUC18 plasmids containing tandem repeats of the Widom-601 sequence with Nucleosome Repeat Lengths (NRL) of 167 or 197 bp. After harvesting and purification using a NucleoBond® Xtra Midi kit (Macherey-Nagel), the plasmids were digested with BseYI and BsaI (New England Biolabs) and purified with a Promega Wizard SV Gel & PCR cleanup kit (Promega). Digoxigenin-11-dUTP (Roche) and Biotin-16-dUTP (Roche) were sequentially built into the ends of the DNA array using LC Klenow Fragment (Thermo Fisher Scientific) and purified with a Promega Wizard SV Gel & PCR cleanup kit (Promega). Chromatin fibers were reconstituted overnight with recombinant human histone octamer (EpiCypher) by salt dialysis in Slide-A-Lyzer 10000 MWCO MINI dialysis tubes (Thermo Scientific) in a titration series of 601-DNA:octamer ratios between 0.8 and 1.5, using a pump to reduce the salt concentration from 5 M NaCl, Tris EDTA (TE) down to TE. Reconstitution quality was checked by native agarose gel electrophoresis.

Flow cell preparation. Flow cells were assembled from 2 cover slips, 24 x 40 mm and 24 x 60 mm, # 1.5 thickness (Menzel Gläser), sandwiched around a Polydimethylsiloxane (PDMS, Sylgard) mold, which was washed thoroughly with milliQ water and 2-propanol. The bottom slide was coated with 0.1% nitrocellulose in amyl-acetate (Ladd Research Industries) prior to incubation with 300 µl of 10 ng/µl sheep anti-digoxigenin (Sigma-Aldrich). Next, the flow cell was passivated overnight with 300 µl 4% (w/v) Bovine Serum Albumin (BSA, Sigma-Aldrich) and 2% (w/v) Tween-20 (Sigma-Aldrich) in milliQ water at 4°C. Experiments were done in measurement buffer (MB): 100 mM KCl, 2 mM $MgCl_2$, 10 mM NaN_3, 10 mM HEPES pH 7.5 and 0.4% (w/v) BSA. Flow cells were washed with MB prior to measurements. 500 µl of 40 pg/µl chromatin in MB was incubated for 10 min at room temperature. Next, 20 µl of M270 magnetic beads (Invitrogen) were washed three times in TE buffer and diluted 500 times in MB before they were flushed into the flow cell. After 10 min the flow cell was washed again with MB and the tethered chromatin fibers were ready for force spectroscopy.

Force spectroscopy on single chromatin fibers using Magnetic Tweezers (MT). A home-built MT, consisting of a 25 Mpix Condor camera (CMOS Vision GmbH), a Camera Link PCIe-1433 frame grabber (National Instruments), a 40x oil NA = 1.3, Plan Fluor objective (Nikon Corporation), an infinity-corrected tube lens (ITL200, Thorlabs), a 100 µW 645 nm LED (IMM Photonics GmbH), a multi-axis piezo scanner P-517.3CL (Physik Instrumente), two 5 mm cube magnets N50 (Supermagnete, Webcraft), a hollow shaft Stepper Motor (Casun) and two M-126.2S1 translation stages (Physik Instrumente). By changing the magnet position between 10 and approximately 0.3 mm above the flow cell bottom at a velocity of ~0.5 mm/s, the force was increased and decreased exponentially between ~0.05 and 70 pN. Typically ~100 beads were tracked in real-time with an accuracy better than 5 nm using a custom tracking algorithm based on Fast Fourier Transform cross-correlation of a 100 pixels region of interest around a bead with a computer generated image featuring cylindrically symmetric pattern of interference signals. Computer controlled magnet movements and synchronous real-time bead tracking were implemented in LabVIEW (National Instruments).

Experimental data analysis. Force-extension traces were selected to discard stuck beads, double tethered beads and other anomalous traces. Drift and offset were subtracted from the curves, such that the extension at forces between 30 and 70 pN followed an extensible Worm Like Chain with a persistence length of 50 nm, a contour length equal to the length of the used DNA substrate and a stretch modulus of 900 pN. Force-Extension data of the fiber at forces below 30 pN were fit to a statistical mechanics model, following Meng et al. (1). The force dependent extension of the chromatin tether was described by the sum of the extension of bare DNA handles, a fraction of stacked nucleosomes, a fraction of partially unwrapped nucleosomes, a fraction of singly-wrapped nucleosomes and a fraction of fully unwrapped nucleosomes. The extension of the stacked fraction was described by a Hookean spring with a rest length of 12 Åper nucleosome for 197 NRL fibers and 6 Å for 167 NRL fibers. The extension of the other nucleosomal conformations followed that of the DNA, in which the contour length equaled

the NRL for the fully unwrapped nucleosome, NRL minus 56 bp for the patially wrapped nucleosome and 5 nm less for the singly-wrapped nucleosome.

The transition between singly wrapped nucleosomes and fully unwrapped nucleosomes was not reversible at the time scale of the experiment as indicated by the experimentally observed hysteresis (data not shown). The unfolding could be reversed though after ~5 s delay at low force between successive pulling curves. These transitions resulted in a characteristic 25 nm steps in extension which directly yielded the number of nucleosomes and tetrasomes in the fiber. All transitions below 10 pN featured no hysteresis and were fitted to an equilibrium model. The free energy of each of the conformations was obtained from integrating the extension of each conformation over the force and was supplemented by a stacking energy ΔG_1, a partial unwrapping energy ΔG_2 and a work term. The free energy contributions of each of the nucleosome conformations, the number of each of the nucleosome conformations and a possible degeneracy term, which describes whether nucleosome pairs unstack independently or cooperatively, were used to calculate the Boltzmann weighted average of the extension of the fiber as a function of force.

Fitting this model, using the above described standard mechanical parameters of DNA, yielded for every force-extension curve the number of nucleosomes, the number of tetrasomes, the stiffness of the fiber per nucleosome, stacking energy ΔG_1, partial unwrapping energy ΔG_2 . The amount of unwrapping $L_{unwrapp}$ = 56 bp and a degeneracy factor D = 0 for 197 NRL fibers and D = 1 for 167 NRL fibers were fixed in all fits and corresponded to previously fitted values(1). All analysis on experimental data was implemented in LabVIEW (National Instruments) and this software is available upon request.

Base pair step parameters. For MC simulations we made use of several modules from the HelixMC package (http://helixmc.readthedocs.io/index.html) described in (2). Base pair step parameters were draw from `helixmc.random_step.RandomStepBase()` using HelixMC file `\data\DNA_gau.npy`. The sequence averaged parameters, as provided in this module, were:

	**Shift (Å)**	**Slide (Å)**	**Rise (Å)**	**Tilt (deg)**	**Roll (deg)**	**Twist (deg)**
mean	0.00	-0.32	3.30	-0.05	1.60	35.21
s.d.	0.57	0.86	0.23	3.56	5.17	6.24

Table S1. Mean and standard deviation of the DNA base step parameters used in HelixMC.

The base pair step parameters and the use of them in MC simulations to reconstruct experimental force-extension data of bare DNA at room temperature and physiological conditions were validated previously (2).

Nucleosome wrapping step parameters. Nucleosome basepair step parameters were extracted from the 1kx5 crystal structure(3) by uploading the pdb file to the web 3DNA website (http://w3dna.rutgers.edu/). From the 14 local minima of the average B-factor in the pdb-file we extracted 14 fixed basepairs: 7, 17, 28, 38, 48, 59, 70, 80, 90, 101, 111, 121, 132, 142. The dyad was defined as the mean base pair index of these 14 fixed locations: base pair 75. A starting conformation of the chromatin fiber was generated by replacing the base pair step parameters of a zero-temperature DNA conformation, by those of the nucleosome. The dyads of each of the nucleosomes defined the NRL of the fiber.

To maintain DNA wrapping around the histone core, we defined step parameters for DNA wrapping relative to the reference frame of the dyad base pair in a similar fashion as for the DNA itself. This yielded the following step parameters for the 14 fixed base pairs relative to the dyad frame:

Base pair	Shift (Å)	Slide (Å)	Rise (Å)	Tilt (deg)	Roll (deg)	Twist (deg)
7	18.8	-13.8	33.7	37	-50	158
17	17.7	-12.5	58.1	60	-79	143
28	2.2	-10.0	79.1	-77	122	-161
38	26.5	-43.9	-63.8	-101	129	93
48	12.8	-9.8	-70.3	-86	102	138
59	4.0	-7.7	-49.5	-35	61	157
70	-0.5	2.7	-16.0	3	-28	-164
80	1.2	-4.4	16.4	-11	9	-170
90	2.2	-10.0	46.5	-30	56	-169
101	2.0	-10.7	68.2	-31	112	-163
111	9.8	-24.0	78.2	-48	150	-149
121	7.8	-21.2	-76.0	-59	139	129
132	2.3	-26.9	-58.3	41	-108	-157
142	2.0	18.2	-39.2	-27	71	174
s.d.	1	1	1	5	5	5

Table S2. Mean and standard deviation of the used DNA wrapping step parameters for each fixed base pair. The mean values were calculated from the 1kx4 crystal structure of the nucleosome. The flexibility, represented by the standard deviations, was guessed.

The unwrapping energy E_{unwrap} was calculated based on a standard deviation of 1 Å for shift, slide and rise and 5 degrees for tilt, roll and twist:

$$E_{unwrap} = \sum_j \min\left(\sum_i 0.5 \frac{k_B T}{{\sigma_{x_i}}^2} \ (x_i - \overline{x_i})^2 \ , E_{unwrap,max} \right) \tag{S1}$$

, with x_i, $\overline{x_i}$, σ_{x_i} and the actual, mean and standard deviation of step parameter i for fixed base pair j and thermal energy $k_B T$. The unwrapping energy was clipped for each fixed base pair at $E_{unwrap,max}$, which was typically set to 2.5 $k_B T$. Because of this clipping, the results were not sensitive for the precise values of the standard deviations of the wrapping step parameters, as long as they were sufficiently small. Note that the interaction potential is harmonic in all 6 degrees of freedom and that we did not include correlations between degrees of freedom in this simple model.

Nucleosome stacking step parameters. Nucleosome stacking was implemented in a similar fashion as base pairs in the rigid base pair model. From the global dimensions of chromatin fibers(4), i.e. a diameter of 330 Å and a nucleosome line density of 20 Å and assuming a left-handed fiber we calculated the following step parameters for nucleosome stacking:

	Shift (Å)	Slide (Å)	Rise (Å)	Tilt (deg)	Roll (deg)	Twist (deg)
mean	44.1	-4.3	85.4	5	55	11
s.d.	1	1	1	5	5	5

Table S3. Mean and standard deviation of the used nucleosome stacking step parameters. The mean values were calculated based on the dimensions of folded chromatin fibers and a left-handed chirality. The flexibility, represented by the standard deviations, was guessed.

We assumed a similar rigidity of the stacking interaction as for wrapping, yielding a similar energy term for each stacked nucleosome:

$$E_{unstack} = \min\left(\sum_i 0.5 \frac{k_B T}{\sigma_{x_i}^2} (x_i - \overline{x_i})^2 \quad , E_{unstack,max} \right) \tag{S2}$$

Simulations of non-interacting nucleosomes were done with $E_{unstack,max}$ = 0. For 1-start and 2-start fibers we evaluated the nucleosome stacking parameters only for neighbor or next-neighbor nucleosomes respectively. Note again that the interaction potential is harmonic in all 6 degrees of freedom and that we did not include correlations between degrees of freedom in this simple model.

Excluded volume effects. Next to attractive interactions between histones and DNA and between nucleosomes we included hard-wall repulsive interactions $E_{rep} = 10^6\ k_B T$ to mimic the experimental conditions:

$$E_{surface/bead} = E_{rep} \tag{S3}$$

if any of the base pair z-coordinate is smaller than 0 or larger than the z-coordinate of the last base pair z_{max}. Following the implementation of the HelixMC package, DNA overlap was not prohibited, which nevertheless resulted in accurate Force-extension curves of bare DNA, even at small forces (see ref 2). Overlap between nucleosomes was penalized however:

$$E_{nuc\ overlap} = E_{rep} \tag{S4}$$

if the center of mass of any pair of nucleosomes was smaller than 55 Å.

Given the short length of the linker DNA, nucleosome-nucleosome exclusion effectively resolved DNA-nucleosome and DNA-DNA overlap due to nucleosome-induced sharp kinks in the DNA trajectory. Accordingly, we did not observe DNA overlap in the resulting structures.***MC initialization.*** A chromatin fiber was initialized by creation of a DNA pose, using `helixmc.pose.HelixPose().`The start conformation, having straight (linker) DNA, represents the zero-temperature structure in absence of nucleosome-nucleosome interactions. Folded fibers were created by setting the stacking energy to infinite and running 1000 MC iterations, as described below. Nucleosome stacking was achieved rapidly, typically within 100 iterations. The initial 1000 conformations were discarded.

MC iterations. After initialization, the stacking energy was clipped to $E_{unstack,max}$. Iterating through every base pair, starting at the first, the step parameters were tentatively replaced by random parameters using `helixmc.random_step.RandomStepSimple()`, drawing from the HelixMC file `\data\DNA_gau.npy`. The change was evaluated and accepted using a standard Metropolis criterion:

$$P(accept) = \begin{cases} 1 & \Delta E \le 0 \\ e^{-\Delta E / k_B T} & \Delta E > 0 \end{cases} \tag{S5}$$

with

$$\Delta E = \Delta E_{unwrap} + \Delta E_{unstack} + \Delta E_{surface/bead} + \Delta E_{nuc\ overlap} - f z_{max}\ . \tag{S6}$$

The latter term represents the work and depends on force f and the z-coordinate of the last base pair z_{max}. Rewrapping of the nucleosomal DNA was achieved by replacing the step parameters of the first free nucleosomal base pair by the corresponding step parameter of the nucleosome crystal structure, rather than from a random draw, and was evaluated using equations S5 and S6. In each iteration the direction of base pair replacements was inverted, resulting in symmetric unwrapping/rewrapping of the nucleosomal DNA.

Simulation settings. All force ramps were performed using 50000 steps in which the force was ramped up and down linearly, up to a preset maximum force. For force-extension curves and 3D animations of the structures, 250 snapshots were sampled in a logarithmic fashion, resulting in an even distribution of the points along the force-extension curve. Typically, a single run of a 1800 bp DNA containing 8 nucleosome fiber took 2-3 days on an Intel® Xeon® CPU E5-26660 v4 @ 2.00GHz PC running Windows 10. Source code is available from GitHub: https://github.com/JvN2/ChromatinMC

Results

Figure S1 shows that fits of individual force-extension curves to the statistical physics model described above yield reproducible parameters that are shared between different NRLs, despite some variation in composition, Like in the experimental data, we analyzed individual force-extension curves separately and refrained from averaging in time or between simulations. All simulations were, however, executed in triplo and featured similar results. Supplemental figure S2 shows the autocorrelation times of the extension and the unwrapping energy of a single time trace at constant force for three different chromatin structures. In all cases correlation is lost after 200 steps, and for the extension 30 steps were sufficient, showing that simulation results were in equilibrium and detailed balance was achieved.

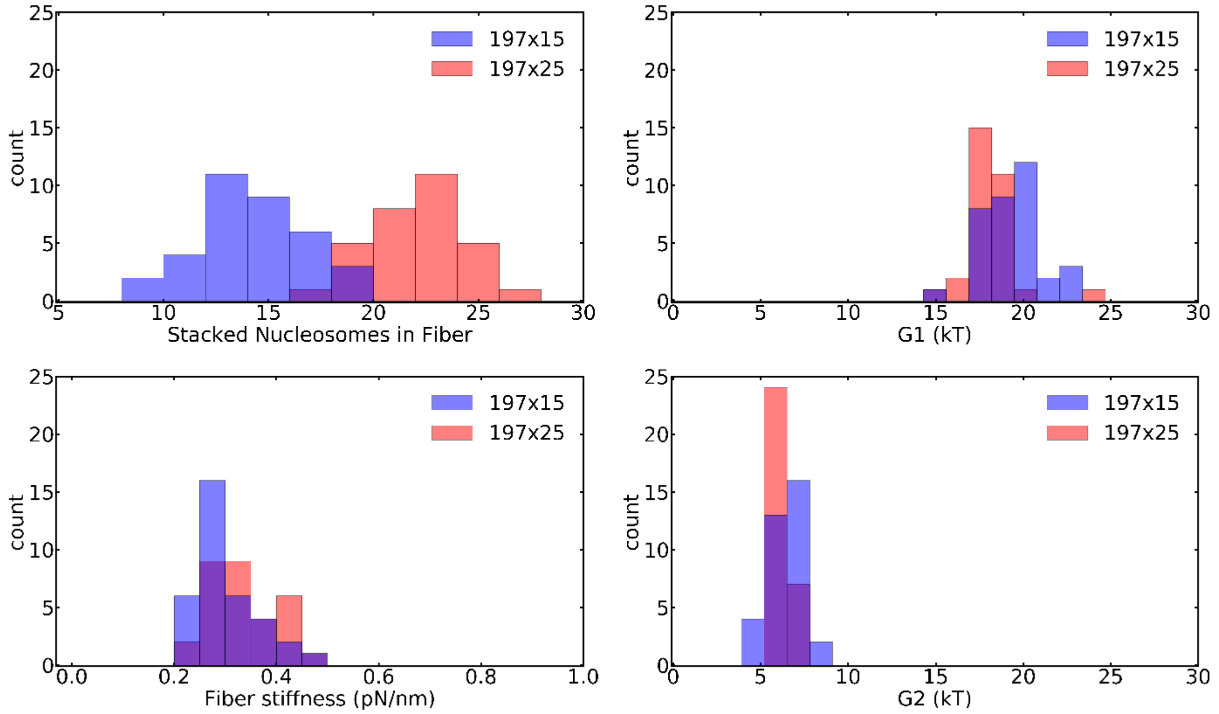

Figure S1. Experimental force extension curves can be captured in 3 parameters that are independent of the number of nucleosomes. Force-extension curves of more than 30 different fibers having either 15 (blue) or 25 (red) 197 NRL repeats of the 601 nucleosome positioning sequence were fitted to the statistical physics model described above with 4 free fitting parameters: a) the number of nucleosomes, b) the stacking energy, c) the fiber stiffness and d) the unwrapping energy up to the singly wrapped nucleosome. Both types of fibers were, on average, slightly under-saturated, but feature a narrow and overlapping distribution in stacking energy, stiffness and residual unwrapping energy, showing that the model parameters fully describe fiber unfolding at forces up to 8 pN.

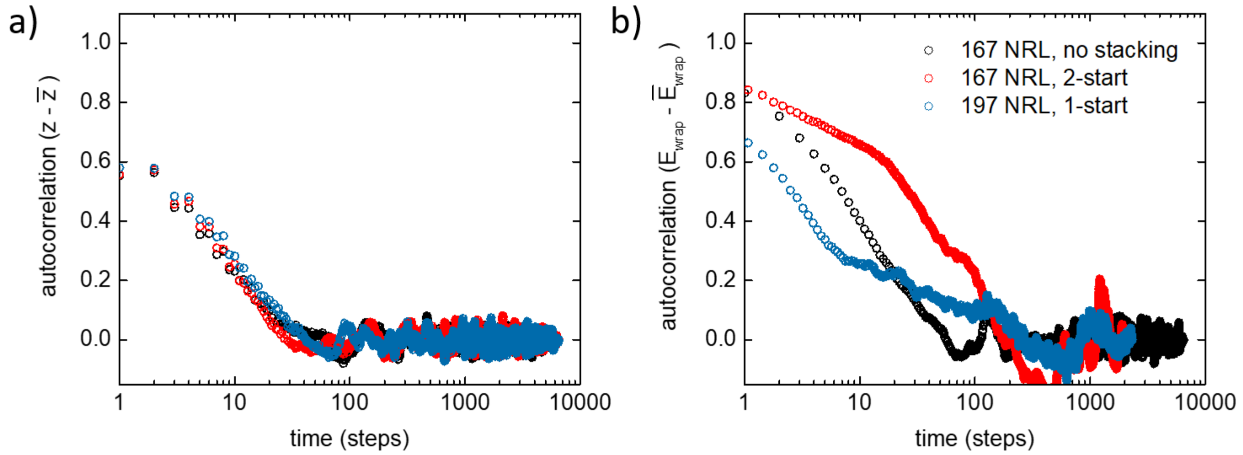

Figure S2. Normalized autocorrelation times for fiber folding. a) The correlation of the extension at f = 0 pN of three typical chromatin fibers is lost after 50 steps in the MC simulations on 4000 bp chromatin fibers containing 15 nucleosomes. The extensions were z = 0.29 ± 0.09 µm, 0.28 ± 0.09 µm and 0.26 ± 0.09 µm (mean ± s.d.) for 167 NRL without stacking (black), 167 NRL in a 2-start structure (red) and 197 NRL in a 1-start structure (blue). b) The corresponding autocorrelation of the unwrapping energy shows that DNA unwrapping is more persistent, but correlations disappear after 70 (no stacking) to 200 steps. The unwrapping energies were E_{unwrap} = 0.7 ± 0.5 k_BT, 0.6 ± 0.3 k_BT and 0.3 ± 0.3 k_BT (mean ± s.d.) for 167 NRL without stacking (black), 167 NRL in a 2-start structure (red) and 197 NRL in a 1-start structure (blue).

References

1. Meng, H., K. Andresen, and J. Van Noort. 2015. Quantitative analysis of single-molecule force spectroscopy on folded chromatin fibers. Nucleic Acids Res. 43.

2. Chou, F., J. Lipfert, and R. Das. 2014. Blind Predictions of DNA and RNA Tweezers Experiments with Force and Torque. 10: 37–47.

3. Luger, K., A.W. Mäder, R.K. Richmond, D.F. Sargent, and T.J. Richmond. 1997. Crystal structure of the nucleosome core particle at 2.8 {Å} resolution. Nature. 389: 251–260.

4. Robinson, P.J.J., L. Fairall, V.A.T. Huynh, and D. Rhodes. 2006. EM measurements define the dimensions of the '"30-nm"' chromatin fiber: evidence for a compact, interdigitated structure. Proc. Natl. Acad. Sci. U. S. A. 103: 6506–6511.
